# 

**Published:** 1868-06

**Authors:** 


### Books and Pamphlets Received.

Circular No. 1—War Department, Surgeon General's Office, Washington, D. C., June 10, 1868. Report on Epidemic Cholera and Yellow Fever in the U. S. Army, in 1867. Lessons in Physical Diagnosis. By Alfred L. Loomis, M. D., Professor of the Institutes and Practice of Medicine, in the Medical Department of the University of New York, etc. New York: Robert M. De Witt, Publisher, No. 13 Frankfort street.

The Institutes of Medicine. By Martin Paine, A. M., M. D., L. L. D. Eighth Edition, Revised. New York: Harper & Brothers, Publishers. 1867.

Twenty-fifth Annual Report of the Managers of the State Lunatic Asylum, for the year 1867.

